# Synchronization Control for Stochastic Neural Networks with Mixed Time-Varying Delays

**DOI:** 10.1155/2014/840185

**Published:** 2014-07-02

**Authors:** Qing Zhu, Aiguo Song, Shumin Fei, Yuequan Yang, Zhiqiang Cao

**Affiliations:** ^1^School of Instrument Science, Southeast University, Nanjing 210096, China; ^2^College of Information Engineering, Yangzhou University, Yangzhou 225009, China; ^3^School of Automation, Southeast University, Nanjing 210096, China; ^4^Institute of Automation, Chinese Academy of Science, Beijing 100190, China

## Abstract

Synchronization control of stochastic neural networks with time-varying discrete and continuous delays has been investigated. A novel control scheme is proposed using the Lyapunov functional method and linear matrix inequality (LMI) approach. Sufficient conditions have been derived to ensure the global asymptotical mean-square stability for the error system, and thus the drive system synchronizes with the response system. Also, the control gain matrix can be obtained. With these effective methods, synchronization can be achieved. Simulation results are presented to show the effectiveness of the theoretical results.

## 1. Introduction

In recent years, stochastic neural networks (NNs) have gained particular research interests. These systems represent a class of stochastic systems that is popular in modeling practical systems which may experience random disturbances and parameters varying. Such a system can be found in biology systems, social systems, and wireless communication networks. Numerous results on stochastic neural network have been reported in the literature [1–5]. As one of the mostly investigated dynamical behaviors, the synchronization in stochastic NNs with or without time delays has drawn significant research interest; see, for example, [6–10] and the references therein.

On the other hand, time delays are frequently encountered in many practical control systems, such as aircraft, chemical, or process control systems. The existence of the time delays may be the source of instability of serious deterioration in the performance of the closed-loop systems. Thus, stability of delayed NNs has been a focal subject for research [11–15]. Most recently, significant and substantial progresses have been achieved in the synchronization of stochastic NNs. These include synchronization of randomly coupled neural networks with Markovian jumping and time-delay [15], adaptive synchronization for stochastic NNs with time-varying delays and distributed delays [16], passivity analysis for discrete time stochastic Markovian jump NNs with mixed time delays [17], adaptive synchronization for stochastic T-S fuzzy NNs with time-delay and Markovian jumping parameters [18], nonfragile synchronization of NNs with time-varying delay and randomly occurring controller gain fluctuation synchronization of biological NN systems with stochastic perturbations and time delays [19], global exponential adaptive synchronization of complex dynamical networks with neutral-type NN nodes and stochastic disturbances [20], adaptive synchronization for uncertain chaotic neural networks with mixed time delays using fuzzy disturbance observer [21], the effects of time delay on the stochastic resonance in feed-forward-loop NN motifs [22], state estimation for wireless network control system with stochastic uncertainty and time delay based on sliding mode observer [23], pinning synchronization in fixed and switching directed networks of Lorenz-type nodes [24], consensus tracking for higher-order multiagent systems with switching directed topologies and occasionally missing control inputs [25], and consensus tracking of multiagent systems with Lipschitz-type node dynamics and switching topologies [26].

Although substantial progresses have been made in the synchronization control of delayed stochastic neural network systems [16–32], there are still some problems which have not been fully studied. For example, many control schemes of time-varying delayed NNs assume that the derivative of the time-delay function is less than one. Also, few works have been done on the synchronization of mixed delayed stochastic NNs. In this paper, we propose sufficient conditions of the synchronization of the stochastic NN system, as well as the state feedback control design. At first, sufficient conditions are proposed in terms of LMIs to guarantee the stochastic asymptotical stability of the error system. The assumption that the derivative of the time-delay function is less than one is eliminated. Then, we give some corollaries to solve the problem in some special cases.

This paper is organized as follows. After the introduction in Section 1, the problem statement and preliminaries are presented in Section 2. Next, some sufficient conditions are presented for the synchronization of the delayed drive and response system. Also, some corollaries and remarks are given to show the advantages of this paper in Section 3. Then, the numerical simulation result is given in Section 4. A conclusion is drawn in Section 5.

## 2. Problem Statement and Preliminaries

Before proceeding, we introduce some notations which will be used later for derivations and discussions. ||·|| denotes the Euclidean norm of a vector or the Frobenius norm of a matrix. *M* > 0 (<0) denotes that matrix *M* is a positive (negative) definite matrix. *E*· denotes the mathematical expectation.

The recurrent network under investigation is

(1) dx(t)=[−Cx(t)+Af(x(t))+Bf(x(t−τ(t))) +D∫t−σ(t)tf(x(s))ds]dt,

where *x*(*t*) = [*x*_1_(*t*), *x*_2_(*t*),…, *x*_*n*_(*t*)]^*T*^ is the system state associated with the neurons, *n* denotes the number of neurons in the network, *f*(*x*(*t*)) = [*f*_1_(*x*_1_(*t*)), *f*_2_(*x*_2_(*t*)),…, *f*_*n*_(*x*_*n*_(*t*))] corresponds to the activation functions of neurons, and *τ*(*t*) and *σ*(*t*) are the time-varying discrete delay and continuous delay, respectively; the initial conditions are given by $x(t)=\phi (t)\in l([-\overset{-}{\tau },0],R^{n})$, where $\phi (t)\in l([-\overset{-}{\tau },0],R^{n})$ denotes the set of all continuous functions from $\left[-\overset{-}{\tau },0\right]$ to *R*. *C* = diag⁡(*c*_1_, *c*_2_,…, *c*_*n*_) is a diagonal matrix, *A* = (*a*_*ij*_)_*n*×*n*_, *B* = (*b*_*ij*_)_*n*×*n*_, and *D* = (*d*_*ij*_)_*n*×*n*_ are the connection weight matrix, discrete time-delay, and continuous time-delay connection weight matrices, respectively.

In this paper, we consider model (1) as the master system. The response system is

(2) dy(t)=[−Cy(t)+Af(y(t))+Bf(y(t−τ(t))) +D∫t−σ(t)tf(y(s))ds+u(t)]dt+σ(t,e(t),e(t−τ(t)))dω(t),

where *A*, *B*, *C*, and *D* are matrices which are the same as (1) and *u*(*t*) is the controller. It has the same structure as the drive system. *e*(*t*) = *y*(*t*) − *x*(*t*) is the error state. *ω*(*t*) = [*ω*_1_(*t*), *ω*_2_(*t*),…, *ω*_*n*_(*t*)]^*T*^ is a *n* dimension Brownian motion defined on a complete probability space (Ω, *F*, *P*).

To propose our main results, it is necessary to make the following assumptions.

(A1) Each function *f*_*i*_ is nondecreasing and globally Lipschitz with a constant *k*_*i*_ > 0:
  (3) $$\begin{array}{c} \left|f_{i}\left(x\right)-f_{i}\left(y\right)\right|\leq k_{i}\left|x-y\right|\;\forall x,y\in R,\;\;i=1,2,\ldots ,n,\\ K=\mathrm{diag}\left(k_{1},k_{2},\ldots ,k_{n}\right). \end{array}$$
(A2) Matrix function *σ*(*t*, *e*(*t*), *e*(*t* − *τ*(*t*))) satisfies
  (4) trace[σT(t,e(t),e(t−τ(t)))σ(t,e(t),e(t−τ(t)))]   ≤||M1e(t)||2+||M2e(t−τ(t))||2,

where *M*_1_ and *M*_2_ are matrices with appropriate dimensions.

(A3) Discrete delay *τ*(*t*) and continuous delay *σ*(*t*) are both differential functions of time, and the following conditions hold:
  (5) $$\begin{array}{c} 0\leq \tau \left(t\right)\leq \overset{-}{\tau },\;\;\dot{\tau }\left(t\right)\leq {\overset{-}{\tau }}_{1},\;\;\;0\leq \sigma \left(t\right)\leq \overset{-}{\sigma }, \end{array}$$

where $\overset{-}{\tau },{\overset{-}{\tau }}_{1}$, and *σ* are positive constants. It is worth to emphasis that the constraint, the derivative of time-delay function is less than one [1], is eliminated in this paper, while a more general upper boundary constraint is utilized instead of it. This improvement makes our results applicable for a wide range of time-delayed stochastic neural networks.

Let error state be *e*(*t*) = *y*(*t*) − *x*(*t*); subtracting (1) from (2) yields the synchronization error dynamical system as follows:

(6) de(t)=[−Ce(t)+Ag(t)+Bg(t−τ(t)) +D∫t−σ(t)tg(s)ds+u(t)]dt+σ(t,e(t),e(t−τ(t)))dω,

where *g*(*t*) = *f*(*y*(*t*)) − *f*(*x*(*t*)).

In this paper, we design a memoryless state feedback controller:

(7) $$\begin{array}{c} u\left(t\right)=Ge\left(t\right), \end{array}$$

where *G* ∈ *R* ^*n*×*n*^ is a constant gain matrix.

Substituting the controller into the error system (6), we get

(8) de(t)=[(−C+G)e(t)+Ag(t)+Bg(t−τ(t)) +D∫t−σ(t)tg(s)ds]dt+σ(t,e(t),e(t−τ(t)))dω.

It is well known that system (8) has a unique solution [33].

**Definition 1 .**

The system (8) is called globally asymptotically mean-square stable, if the following holds:

(9) $$\begin{array}{c} \underset{t\to \infty }{\lim}E{||e\left(t\right)||}^{2}=0,\;\text{for}\;\;\text{any}\;\;e\left(t_{0}\right), \end{array}$$

where *E*· is the mathematical expectation. The following Lemma is given.

**Lemma 2 (Schur complement lemma).**

For matrices *A*, *B*, and *C* with compatible dimensions, the following three conditions are equivalent:

(10) $$\begin{array}{c} \;\left(a\right)\;\left[\begin{array}{cc} A & C\\ C^{T} & B \end{array}\right]<0; \end{array}$$

(11)  (b) A−CB−1CT<0,  B<0,   if  B  is  invertible;

(12)  (c) B−CTA−1C<0,  A<0, if  A  is  invertible.

**Lemma 3 .**

For vector function *f*(*t*) ∈ *R* ^*n*^ and symmetric matrix *P* > 0, the following holds:

(13) $$\begin{array}{c} \overset{b}{\underset{a}{\int }}f^{T}\left(t\right)dt\;P\overset{b}{\underset{a}{\int }}f\left(t\right)dt\leq \left(b-a\right)\overset{b}{\underset{a}{\int }}f^{T}\left(t\right)Pf\left(t\right)dt. \end{array}$$

## 3. Criteria of Synchronization

In this section, new criteria are presented for the global asymptotical stability of the equilibrium point of the neural network defined by (8), and thus the drive system (1) synchronizes with the response system (2). Its proof is based on a new Lyapunov functional method and linear matrix inequality (LMI) approach.

**Theorem 4 .**

Under the assumptions A1–A3, the equilibrium point of model (8) is called globally asymptotically stable in mean-square, if there exist symmetric positive-definite matrices *P*_*j*_  ( *j* = 1,…, 4), diagonal matrices *D*_1_, *D*_2_, and general matrix *Q*_1_ such that the following inequalities hold:

(14) Π=[Π11Q1Π13Π140P1D+τ−2Dτ−(−C+G)TP1∗Π220KTD2−Q100∗∗Π33τ−2ATP1B0τ−2ATP1D0∗∗∗Π440τ−2BTP1D0∗∗∗∗−P100∗∗∗∗∗−P4+τ−2DTP1D0∗∗∗∗∗∗−P1]<0,

(15) P1<ρI,

where

(16) Π11=P1(−C+G)+(−C+G)TP1+P2+ρM1TM1,Π13=P1A+KTD1+τ−2(−C+G)TP1A,Π14=P1B+τ−2(−C+G)TP1B,Π22=−(1−τ−1)P2−Q1−Q1T+ρM2TM2,Π33=P3−2D1+σ−2P4+τ−2ATP1A,Π44=−(1−τ−1)P3−2D2+τ−2BTP1B.

**Proof**

The Lyapunov function candidate is given as

(17) $$\begin{array}{c} V\left(t\right)=\overset{5}{\underset{i=1}{\sum }}{\;V}_{i}\left(t\right), \end{array}$$

where

(18) V1(t)=eT(t)P1e(t),V2(t)=∫t−τ(t)teT(s)P2e(s)ds,V3(t)=∫t−τ(t)tgT(s)P3g(s)ds,V4(t)=τ−∫0τ−∫t−ste˙T(θ)P1e˙(θ)dθ ds,V5(t)=σ−∫−σ−0∫t+stgT(θ)P4g(θ)dθ ds.

The weak infinitesimal operator *L* of the process $\left\{x_{t}=x(t+s),t\geq 0,-\overset{-}{\tau }\leq t\leq 0\right\}$ is given by

(19) LV1=2eT(t)P1[(−C+G)e(t)+Ag(t)     +Bg(t−τ(t))+D∫t−σ(t)tg(s)ds]+trace[σT(t,e(t),e(t−τ(t)))    ×P1σ(t,e(t),e(t−τ(t)))].

By (5) and (14), we have

(20) trace[σT(t,e(t),e(t−τ(t)))     ×P1σ(t,e(t),e(t−τ(t)))] ≤ρ trace[σT(t,e(t),e(t−τ(t)))       ×σ(t,e(t),e(t−τ(t)))] =ρ[eT(t)M1TM1e(t)   +eT(t−τ(t))M2TM2e(t−τ(t))],

(21) LV2(t)=eT(t)P2e(t)−(1−τ˙(t))eT×(t−τ(t))P2e(t−τ(t)),

(22) LV3(t)=gT(t)P3g(t)−(1−τ˙(t))gT×(t−τ(t))P3g(t−τ(t)),

(23) $$\begin{array}{c} LV_{4}\left(t\right)={\overset{-}{\tau }}^{2}{\dot{e}}^{T}\left(t\right)P_{1}\dot{e}\left(t\right)-\overset{-}{\tau }\overset{t}{\underset{t-\overset{-}{\tau }}{\int }}{\dot{e}}^{T}\left(s\right)P_{1}\dot{e}\left(s\right)ds,‍ \end{array}$$

(24) $$\begin{array}{c} LV_{5}\left(t\right)={\overset{-}{\sigma }}^{2}g^{T}\left(t\right)P_{4}g\left(t\right)-\overset{-}{\sigma }\overset{t}{\underset{t-\overset{-}{\sigma }}{\int }}g^{T}\left(s\right)P_{4}g\left(s\right)ds. \end{array}$$

By Lemma 3, we obtain

(25) −τ−∫t−τ−te˙T(s)P1e˙(s)ds  ≤−τ(t)∫t−τ(t)te˙T(s)P1e˙(s)ds  ≤−∫t−τ(t)te˙T(s)ds P1∫t−τ(t)te˙(s)ds,

(26) −σ−∫t−σ−tgT(s)P4g(s)ds  ≤−σ(t)∫t−σ(t)tgT(s)P4g(s)ds  ≤−∫t−σ(t)tgT(s)ds P4∫t−σ(t)tg(s)ds.

By Newton-Leibniz formula, we have

(27) $$\begin{array}{c} e\left(t\right)-e\left(t-\tau \left(t\right)\right)=\overset{t}{\underset{t-\tau (t)}{\int }}\dot{e}\left(s\right)ds. \end{array}$$

From assumption A1, we get

(28) $$\begin{array}{c} g^{T}\left(t\right)D_{1}g\left(t\right)\leq g^{T}\left(t\right)D_{1}Ke\left(t\right),\\ g^{T}\left(t-\tau \left(t\right)\right)D_{2}g\left(t-\tau \left(t\right)\right)\leq g^{T}\left(t-\tau \left(t\right)\right)D_{2}Ke\left(t-\tau \left(t\right)\right), \end{array}$$

where *K*, *D*_1_, and *D*_2_ are positive-definite diagonal matrices. By (27) and (28), we define several nonnegative expressions as follows:

(29) L1=2e(t−τ(t))Q1[e(t)−e(t−τ(t))−∫t−τ(t)te˙(s)ds]=0,

(30) $$\begin{array}{c} L2=2\left[g^{T}\left(t\right)D_{1}Ke\left(t\right)-g^{T}\left(t\right)D_{1}g\left(t\right)\right]\geq 0, \end{array}$$

(31) L3=2[gT(t−τ(t))D2Ke(t−τ(t))−gT(t−τ(t)) ×D2g(t−τ(t))]≥0.

Then expressions *L*1, *L*2, and *L*3 are to be added to the inequality of *LV*(*t*) to facilitate the proof.

Therefore, combining (19)–(29) we have

(32) LV(t)≤∑i=15 LVi(t)+L1+L2+L3≤eT(t)[2P1(−C+G)+ρM1TM1+P2]e(t)+eT(t−τ(t))[−(1−τ−1)P2+ρM2TM2−2Q1]×e(t−τ(t))+gT(t)[P3−2D1+σ−2P4]g(t)+gT(t−τ(t))[−(1−τ−1)P3−2D2]g(t−τ(t))−∫t−τ−te˙T(s)ds P1∫t−τ−te˙(s)ds−∫t−σ−tgT(θ)dθ P4∫t−σ−tg(θ)dθ+2eT(t)Q1e(t−τ(t))+eT(t)[2P1A+2KTD1]g(t)+2eT(t)[P1B]g(t−τ(t))+2eT(t)[P1D]∫t−σ(t)tg(θ)dθ+2eT(t−τ(t))[KTD2]g(t−τ(t))+2eT(t−τ(t))[Q1]∫t−τ−te˙(s)ds+[(−C+G)e(t)+Ag(t)+Bg(t−τ(t))  + D∫t−σ(t)tg(s)ds]T×τ−2P1[(−C+G)e(t)+Ag(t)    +Bg(t−τ(t))+D∫t−σ(t)tg(s)ds]≤ξTNξ,

where

(33) ξ=[eT(t),eT(t−τ(t)),gT(t),gT(t−τ(t)), ∫t−τ(t)te˙T(s)ds,∫t−σ(t)tgT(s)ds]T,N=[N11Q1Π13Π140P1D+τ−2D∗Π220KTD2−Q10∗∗Π33τ−2ATP1B0τ−2ATP1D∗∗∗Π440τ−2BTP1D∗∗∗∗−P10∗∗∗∗∗−P4+τ−2DTP1D],N11=P1(−C+G)+(−C+G)TP1+P2+ρM1TM1+τ−2(−C+G)TP1(−C+G).

We introduce matrices *M* and *H* which satisfy

(34) $$\begin{array}{c} \Pi =\left[\begin{array}{cc} M & H\\ H^{T} & -P_{1} \end{array}\right],\;\;N=M-H{\left(-P_{1}\right)}^{-1}H^{T}, \end{array}$$

where

(35) M=[Π11Q1Π13Π140P1D+τ−2D∗Π220KTD2−Q10∗∗Π33τ−2ATP1B0τ−2ATP1D∗∗∗Π440τ−2BTP1D∗∗∗∗−P10∗∗∗∗∗−P4+τ−2DTP1D],H=[τ−(−C+G)TP100000].

By Lemma 2, we get that Π < 0⇔*N* < 0, −*P*_1_ < 0. Thus *LV* < 0. From (24) and the Ito formula, it is obvious that

(36) $$\begin{array}{c} EV\left(t\right)-EV\left(t_{0}\right)=E\overset{t}{\underset{t_{0}}{\int }}LV\left(s\right)ds. \end{array}$$

From the definition of *V*(*t*) in (15), there exists positive constant *λ*_1_ such that

(37) λ1E||e(t)||2≤EV(t)≤EV(t0)+E∫t0tLV(s)ds≤EV(t0)+λmax⁡E∫t0t||e(s)||2ds,

where *λ*_max⁡_ is the maximal eigenvalue of *N* and it is negative.

Therefore, from (26) and the discussion in [34], we know that the equilibrium of (11) is globally asymptotically stable in mean-square. This completes the proof.

**Corollary 5 .**

Under the assumptions A1–A3, the equilibrium point of model (8) is called globally asymptotically stable in mean-square, if there exist symmetric positive-definite matrices *P*_*j*_  (*j* = 1,…, 4), diagonal matrices *D*_1_, *D*_2_, and general matrices *Q*_1_ and *W*, such that the following inequalities hold:

(38) Π=[Π11Q1Π13Π140P1D+τ−2Dτ−(−CTP1+WT)∗Π220KTD2−Q100∗∗Π33τ−2ATP1B0τ−2ATP1D0∗∗∗Π440τ−2BTP1D0∗∗∗∗−P100∗∗∗∗∗−P4+τ−2DTP1D0∗∗∗∗∗∗−P1]<0,P1<ρI,

where

(39) Π11=−P1C−CTP1+W+WT+P2+ρM1TM1,Π13=P1A+KTD1+τ−2(−CTP1+WT)A,Π14=P1B+τ−2(−CTP1+WT)B,Π22=−(1−τ−1)P2−Q1−Q1T+ρM2TM2,Π33=P3−2D1+σ−2P4+τ−2ATP1A,Π44=−(1−τ−1)P3−2D2+τ−2BTP1B.

Furthermore, the control gain matrix *G* is given as

(40) $$\begin{array}{c} G=P_{1}^{-1}W. \end{array}$$

**Proof**

Let *W* = *P*_1_ *G* in Theorem 4; it is easy to get the result.

**Corollary 6 .**

If the systems (1) and (2) have no integral time-delay items (e.g., *D* = 0), the result can be simplified as follows. Under assumptions A1–A3 and *D* = 0, the equilibrium point of model (8) is called globally asymptotically stable in mean-square, if there exist symmetric positive-definite matrices *P*_*j*_  (*j* = 1,…, 4), diagonal matrices *D*_1_, *D*_2_, and general matrices *Q*_1_ and *W*, such that the following inequalities hold:

(41) Π=[Π11Q1Π13Π140τ−(−CTP1+WT)∗Π220KTD2−Q10∗∗Π33τ−2ATP1B00∗∗∗Π4400∗∗∗∗−P10∗∗∗∗∗0∗∗∗∗∗−P1]<0,P1<ρI,

where

(42) Π11=−P1C−CTP1+W+WT+P2+ρM1TM1,Π13=P1A+KTD1+τ−2(−CTP1+WT)A,Π14=P1B+τ−2(−CTP1+WT)B,Π22=−(1−τ−1)P2−Q1−Q1T+ρM2TM2,Π33=P3−2D1+σ−2P4+τ−2ATP1A,Π44=−(1−τ−1)P3−2D2+τ−2BTP1B.

Furthermore, the control gain matrix *G* is given as

(43) $$\begin{array}{c} G=P_{1}^{-1}W. \end{array}$$

**Proof**

Let *D* = 0 in system (8). It is much similar to the proof of Theorem 4 and is then omitted.

**Remark 7 .**

In this paper, we use a memoryless state feedback control to achieve the synchronization of the stochastic delayed neural network system. Compared with the delayed feedback control [1], it is easy to implement. The biggest advantage of this approach is that we only need to know the boundaries of time delays, instead of exact values of time delays.

**Remark 8 .**

In Corollary 5, we give an approach to choose the control gain matrix *G* and it is helpful for the design of the controller to let the drive system synchronize with the response system.

## 4. Numerical Simulations

In this section, we present numerical simulations to show the effectiveness of the theoretical results.

Consider the drive system (1) of a delayed Hopfield neural network with coefficient matrices as

(44) A=[1−0.5−0.21],  B=[−0.5−0.1−0.3−0.6],C=[1001],  D=[1001],τ(t)=0.3+0.3sin(4t),  σ(t)=0.4−0.2sin(t),f(t)=[tanh⁡(x1)tanh⁡(x2)].

The corresponding response system refers to (2), where

(45) $$\begin{array}{c} \sigma \left(t,e\left(t\right),e\left(t-\tau \left(t\right)\right)\right)=\left[\begin{array}{cc} ||e\left(t\right)|| & 0\\ 0 & 0.6||e\left(t-\tau \left(t\right)\right)|| \end{array}\right]. \end{array}$$

It is easy to see that *K* = *M* = *M*_1_ = *I*, where *I* is the identity matrix. By Theorem 4 and Corollary 5, we can get the following feasible solutions:

(46) $$\begin{array}{c} W=\left[\begin{array}{cc} -1918.2 & 12.4\\ 77.5 & -1984.4 \end{array}\right],\\ Q_{1}=\left[\begin{array}{cc} 545.5568 & 3.6943\\ 17.0650 & 557.7364 \end{array}\right],\\ D_{1}=\left[\begin{array}{cc} 1046.5 & 0\\ 0 & 1207.4 \end{array}\right],\\ D_{2}=\left[\begin{array}{cc} 226.3259 & 0\\ 0 & 242.7899 \end{array}\right],\\ \rho =1084.8,\;\;G=\left[\begin{array}{cc} -1.8352 & 0.0425\\ 0.1038 & -1.9002 \end{array}\right],\\ P_{1}=\left[\begin{array}{cc} 1.046.2 & 0.016.9\\ 0.016.9 & 1.044.7 \end{array}\right],\;\;P_{2}=\left[\begin{array}{cc} 52.1765 & -18.3810\\ -18.3810 & 48.2007 \end{array}\right],\\ P_{3}=\left[\begin{array}{cc} 94.2132 & -39.7350\\ -39.7350 & 86.2442 \end{array}\right],\;\;P_{4}=\left[\begin{array}{cc} 2626.1 & 148.4\\ 148.4 & 2760.3 \end{array}\right]. \end{array}$$

The initial states of drive system and response system are *x*(0) = [0.75  0]^*T*^ and *y*(0) = [0.5  0.1]^*T*^.

**Figure 1:**
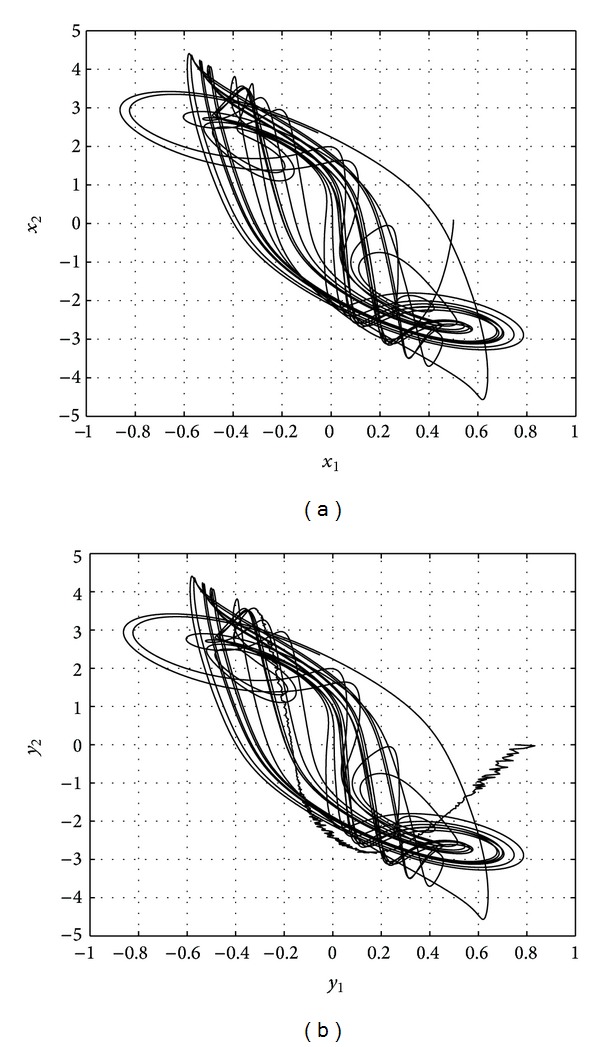
Phase trajectories of drive (a) and response (b) system.

**Figure 2:**
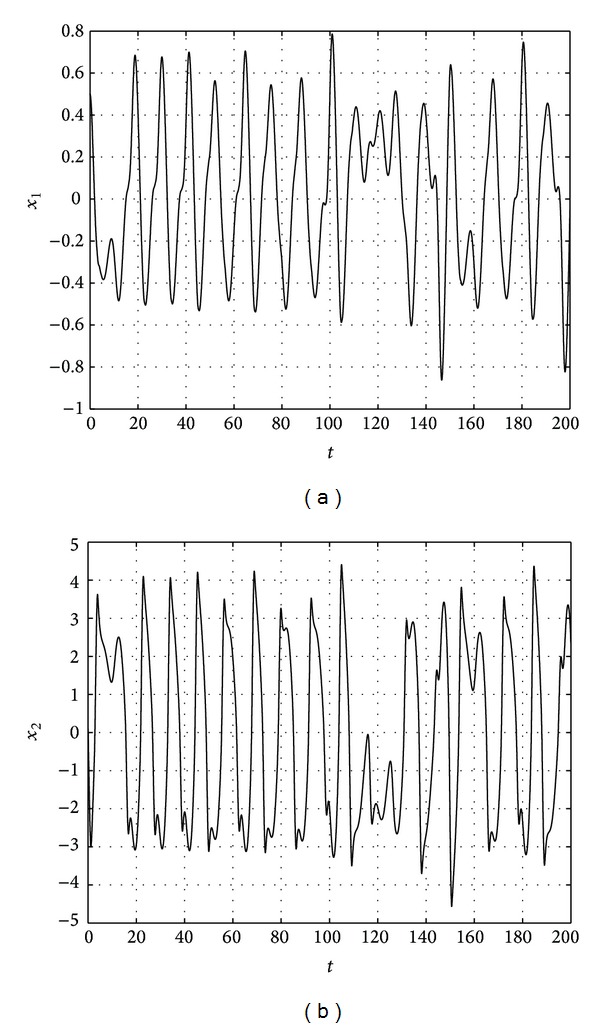
State trajectories of drive system.

**Figure 3:**
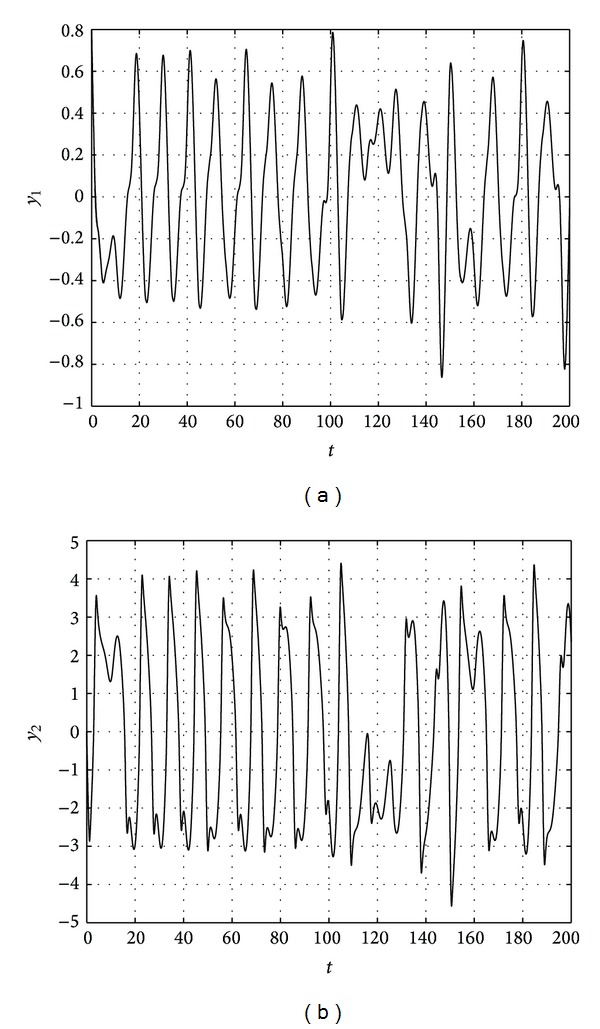
State trajectories of response system.

**Figure 4:**
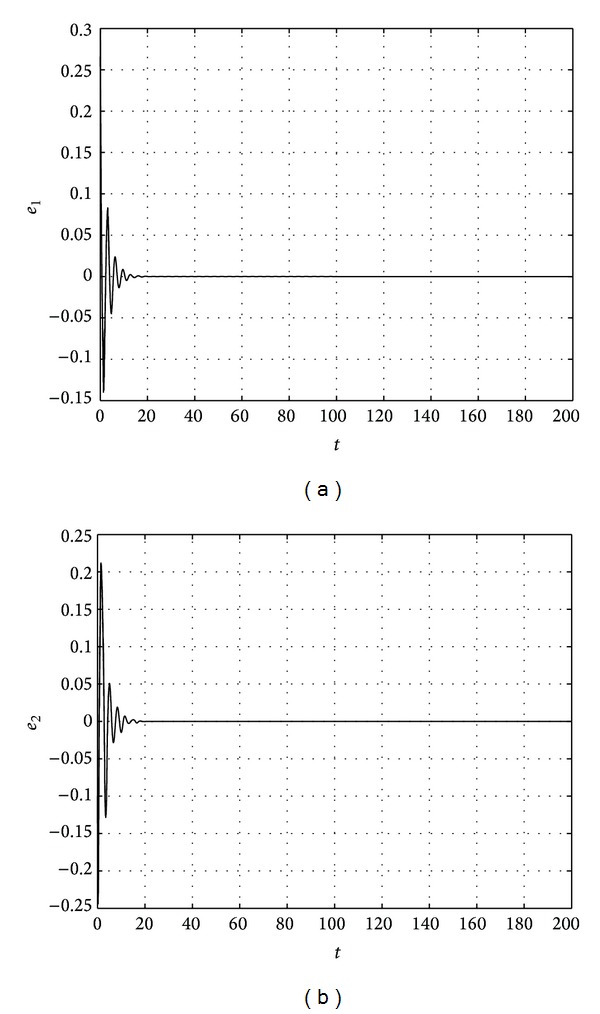
State trajectories of error system.

The results are shown in Figures 1, 2, 3, and 4.

## 5. Conclusion

In this paper, we considered synchronization control of stochastic neural networks with time-varying delays. We use Lyapunov functional method and linear matrix inequality (LMI) technique to solve this problem. Several sufficient conditions have been derived to ensure the global asymptotical stability for the error system, and thus the drive system synchronizes with the response system. Also, the control gain can be obtained. The results are novel since there are few works about the synchronization of mixed delayed system and the constraint of the derivative of the time-delay function is relaxed. It is easy to apply these sufficient conditions to the real networks. Finally, a numerical simulation is presented to verify the theoretical results.
